# Supplementary material for: Cooperation between Public Primary Health Care and Occupational Health Care Professionals in Work Ability-Related Health Issues
Source: Int J Environ Res Public Health. 2022 Sep 21;19(19):11916. doi: 10.3390/ijerph191911916 (PMC9564539; doi:10.3390/ijerph191911916)
Supplement: Supplementary file 1 [file ijerph-19-11916-s001.zip › Supplementary document S1.pdf]

Semi structured interview guide for public primary health care and occupational health care professionals

Professionals from public primary health care and occupational health care were asked the same questions.

BACKGROUND

What is your profession?

How long have you been working on health care services?

How much previous experience do you have from working in similar profession?

Have you previously worked on any another health care sector?

HOW PATIENTS WORK DISABILITY AND WORK DISABILITY RISK ARE PERCEIVED IN PUBLIC PRIMARY HEALTH CARE AND OCCUPATIONAL HEALTH CARE – WHO ARE THEY?

What kind of work ability issues you face in your job?

Estimate, how often you face work ability issues?
Kuinka usein työssäsi kohtaat työkykyasioita?

Describe, how you assess the patients' work ability in practice?

What do you think; when the patient is unable to work?

Have you noticed patients to use public primary health care services, occupational health care services and special health care services simultaneously or do patients treat the same medical condition simultaneously on multiple sectors?

Describe, what kind of health conditions/issues patients usually visit doctors on multiple sectors?

Are some profession or patient groups overrepresented in those patients who would require workability support?

How about according to age, gender, medical diagnosis, labour market position, profession or by some other factor?

If the interviewee describes gender differences, for example women and mental health: Why do you think so: don't men have, or don't they wish to discuss their mental problems?

Do unemployed and employed differ in regard of their work ability?

Do they differ aaccording to age, gender, medical diagnosis, labour market position, profession or by some other factor?

FOLLOW-UP MEASURES AND SERVICE USE

Do you know, what kind of work ability support services are available for patients?

> *Work ability support services include for example occupational health negotiations between occupational health services and workplace, changes at workplace, rehabilitation groups etc.*
>
> *Do you think that there are enough work ability support services available?*

How is the patient with compromised work ability or with decreased work ability guided forward in your organization?

How are the work ability support services carried out in practice?

> *What stakeholders primarily provide work ability support services? What are those services?*

How well does the work ability support services function from the health care professionals' perspective?

> *How would you estimate the resources that work ability support services have?*
>
> *Should the current work ability support services be somehow changed or developed?*

Do you know, how patients regard the work ability support services?

> *How could patients' commitment to the occupational health services be improved?*
>
> *Do you think that work ability support services are easy for patients to use?*

Have you guided patients to work ability support services?

> *How did you feel about it?*

Would you like to receive support or education for guiding patients into work ability support services?

> *Or education about work ability assessment?*
>
> *What kind support or education would you prefer?*

THE COOPERATION OF PUBLIC PRIMARY HEALTH CARE AND OCCUPATIONAL HEALTH SERVICES

How would you describe the cooperation between public primary health care and occupational health care services in your own organization?

Do you have mutually agreed cooperation practices with occupational health care services / public primary health care services?

If has: Do you think, is there anything that should be developed in the cooperation practises?

If not: Do you think that your organization should have mutually agreed cooperation practices with occupational health care services / public primary health care?

How people in your organization generally regard the cooperation between public primary health care and occupational health care services?

Is there anything to be improved in the cooperation?

Is there anything that you would like to add, something that I didn’t ask?
