# Supplementary figures and images for: Cooperation between Public Primary Health Care and Occupational Health Care Professionals in Work Ability-Related Health Issues
Source: Int J Environ Res Public Health. 2022 Sep 21;19(19):11916. doi: 10.3390/ijerph191911916 (PMC9564539; doi:10.3390/ijerph191911916)

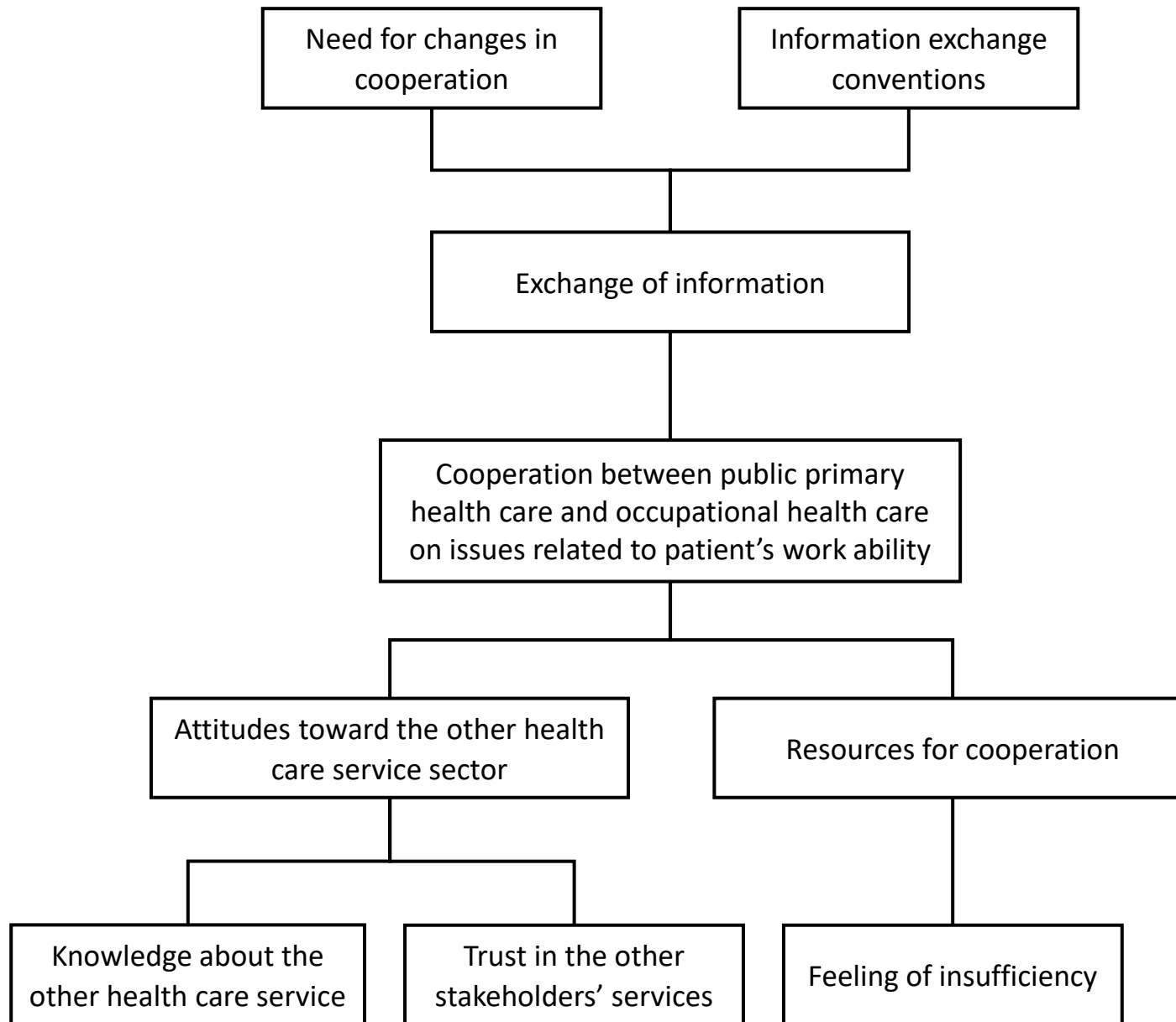

Supplement: Supplementary file 1 [file ijerph-19-11916-s001.zip › Supplementary Figure S1.pdf]
